# Chewing Efficiency, Global Cognitive Functioning, and Dentition: A Cross-sectional Observational Study in Older People With Mild Cognitive Impairment or Mild to Moderate Dementia

**DOI:** 10.3389/fnagi.2020.00225

**Published:** 2020-09-09

**Authors:** Suzanne Delwel, Andrea B. Maier, Donya Parvaneh, Jesse Meijers, Erik J. A. Scherder, Frank Lobbezoo

**Affiliations:** ^1^Department of Clinical Neuropsychology, Faculty of Behavioral and Movement Sciences, Vrije Universiteit Amsterdam, Amsterdam, Netherlands; ^2^Department of Orofacial Pain and Dysfunction, Academic Centre for Dentistry Amsterdam (ACTA), University of Amsterdam and Vrije Universiteit Amsterdam, Amsterdam, Netherlands; ^3^Department of Medicine and Aged Care, The Royal Melbourne Hospital, University of Melbourne, Melbourne, VIC, Australia; ^4^Department of Human Movement Sciences, Faculty of Behavioural and Movement Sciences, Amsterdam Movement Sciences, Vrije Universiteit Amsterdam, Amsterdam, Netherlands; ^5^Independent Researcher, Amsterdam, Netherlands

**Keywords:** mastication, chewing, cognitive impairment, cognitive dysfunction, dementia, aged, geriatric dentistry, gerodontology

## Abstract

**Introduction**: Previous studies suggest an association between poor mastication and cognitive impairment. The role of chewing efficiency and dentition in this relation is unclear. The aim was to examine global cognitive functioning and dentition as predictors for chewing efficiency, in older people with mild cognitive impairment (MCI) or dementia.

**Methods**: In this observational cross-sectional study, 136 people with MCI or dementia were included. The chewing efficiency was assessed with a two-colored chewing gum and analyzed with the Chewing Efficiency Analysis software. The level of global cognitive functioning was measured with the Mini Mental State Examination (MMSE) by trained clinical staff. An oral examination was performed by a dentist and included the number of present teeth, the number of occluding pairs, and the presence of prostheses. Age, gender, and educational years were derived from the medical records. Univariate and multivariate backward stepwise linear regression analyses were used to evaluate global cognitive functioning and dentition as predictors for chewing efficiency.

**Results**: The mean age of the participants was 82.1 (SD 5.8) years, and 74 (54.4%) were female. The participants had a median MMSE score of 22.4 (IQR 18.0–26.0) and a median Chewing Efficiency Analysis score of 0.46 (IQR 0.14–0.59). The median number of teeth was 13.0 (IQR 0.0–23.0), and the median number of occluding pairs was 0.0 (IQR 0.0–7.0). Sixty-four (47.4%) of the participants wore full prosthesis in the upper jaw. In univariate linear regression analyses, predictive factors for the Chewing Efficiency Analysis score were age, MMSE score, full prosthesis in the upper jaw, number of present teeth, and number of occluding pairs. In the multivariate model, full prosthesis in the upper jaw and number of occluding pairs were significant predictors for the Chewing Efficiency Analysis score. Participants with full prosthesis in the upper jaw had a lower Chewing Efficiency Analysis score than participants with natural dentition in the upper jaw.

**Conclusion**: Better mastication is associated with a higher number of occluding pairs. Full prosthesis in the upper jaw is related to a lower chewing efficiency. Global cognitive functioning is not associated with mastication in older people with MCI or mild-to-moderate dementia. This might be explained by sufficient capacity for compensation of reduced mastication in this group.

## Introduction

Worldwide, around 48 million people have dementia, and this number is estimated to increase to 1.25 billion by 2050 (Prince et al., 2013). Dementia is a clinical syndrome, characterized by memory loss and impairment in language (aphasia or dysphasia), motor function (apraxia), visual recognition (agnosia), and executive functioning, resulting in difficulties during activities in daily life (Kester and Scheltens, 2009). Mild cognitive impairment (MCI) is characterized by a mild impairment of cognition, more than can be expected from age alone, at which daily functioning is largely unaffected (Petersen et al., 2014). The prevalence of MCI increases with age and varies between 6.7% in people aged 60–64 years and 25.2% for 80–84 years (Petersen et al., 2018). Older people with MCI are at higher risk for developing dementia, among which Alzheimer’s disease (AD; Petersen et al., 2018).

At the same time, currently aging individuals maintain their natural dentition until a higher age as a result of better prevention of oral disease and improved professional dental care (Petersen, 2003). However, throughout an individual’s life, teeth are lost, mainly as a result of caries and periodontitis (Tonetti et al., 2017), especially in people with dementia, who show a decrease in oral hygiene self-care and professional dental care utilization (Fereshtehnejad et al., 2017). Interestingly, several studies examined the relation between periodontitis, tooth loss, and cognitive impairment (Wu et al., 2016; Delwel et al., 2017, 2018; Tonsekar et al., 2017). Although some of these studies indicate such a relation, the combined results are inconclusive (Wu et al., 2016; Tonsekar et al., 2017). Most of the studies concerning cognition and mastication used the number of present teeth as a measure for mastication (Tada and Miura, 2017). Only a few studies included an objective assessment of chewing efficiency for mastication, such as a mixing ability test (Kimura et al., 2013; Elsig et al., 2015; Weijenberg et al., 2015).

The aim of the current study was to examine global cognitive functioning and dentition as predictors for chewing efficiency, in older people with MCI or dementia. Dentition was represented by the number of teeth, occluding pairs, and the presence of prosthesis.

## Materials and Methods

### Study Design, Setting, and Participants

In this cross-sectional study, the relation between chewing efficiency, global cognitive functioning, and dentition was examined in participants with MCI or dementia. The participants were recruited at the geriatric outpatient clinics of the Amsterdam UMC and the Amstelland Hospital in Amstelveen and the psychogeriatric wards of ten nursing homes in Amsterdam and the surrounding area, as part of the Pain in Dementia Amsterdam, or PainDemiA study. The study protocol was approved by the Medical Ethics Review Committee of the Amsterdam UMC (approval number NL 43861.029.13) and was described elsewhere (van Kooten et al., 2015). The study protocol article describes the inclusion and exclusion criteria, power calculation, and procedure to establish the dementia diagnosis. Participants who met the following criteria included: aged 60 or older; diagnosis of MCI or dementia, i.e., AD, vascular dementia (VaD), frontotemporal dementia (FTD), and dementia with Lewy bodies (DLB); and a signed informed consent by the participant or legal representative. In the outpatient memory clinics at the hospitals, the MCI or dementia diagnosis was established by a multidisciplinary team of medical doctors, nurses, neuropsychologists, and neurologists, based on the National Institute of Neurological and Communicative Disorders and Stroke (NINCDS) and the AD and Related Disorders Association (ADRDA) criteria for Alzheimer dementia (McKhann et al., 2011), the National Institute of Neurological Disorders and Stroke Association (NINDS) and Association Internationale pour la Recherché et l’Enseignement en Neurosciences (AIREN) criteria for vascular dementia (Román et al., 1993), the revised criteria for FTD (Rascovsky et al., 2011), the revised criteria for DLB (McKeith et al., 2005), and the revised criteria for MCI (Petersen et al., 2014). At the nursing homes, the formal dementia diagnosis in the medical chart was used, which was usually based on the Diagnostic and Statistical Manual of Mental Disorders (DSM-IV) criteria for dementia (American Psychiatric Association, 2000). Participants without a MCI or dementia diagnosis were excluded. The data was collected between April 2014 and December 2015, in accordance with the STrengthening the Reporting of OBservational Studies in Epidemiology (STROBE) statement (Sanderson et al., 2007). The demographic characteristics, gender, date of birth, and educational status were derived from the medical records of the participants.

### Global Cognitive Functioning

The global cognitive functioning of the participants was measured with the Mini Mental State Examination (MMSE) by trained clinical staff at the outpatient clinics of the hospitals and a neuropsychologist at the nursing homes (Folstein et al., 1975). The MMSE screens different cognitive domains: orientation in time and place (10 points), immediate recall (3 points), attention and calculation (5 points), delayed recall (3 points), language (8 points), and visual construction (1 point). The minimum score is 0, and the maximum total score is 30. Participants with an MMSE score of lower than 14 points were excluded, because understanding of the instructions could not be assumed (Hadjistavropoulos et al., 2010).

### Chewing Efficiency

The chewing efficiency was tested with a two-colored chewing gum, consisting of blue and pink Bubblicious^®^ bubble gum (Cadbury Nederland B.V., Breda, Netherlands). The participants were instructed to chew on the two-colored chewing gum as normally as possible for 1 min. After 60 s, the dentist asked the participants to return the chewing gum, after which it was placed between two clear cellophane sheets to be flattened between two connected acrylic plates. Thereafter, the flattened chewing gum was photographed in a standard setting with a digital Canon 450D^®^ camera (Canon Inc., Tokyo, Japan; Weijenberg et al., 2013).

The chewing gum photos were analyzed with the Chewing Efficiency Analysis software, according to the algorithm described in Table 1. The minimum Chewing Efficiency Analysis score was 0.00, and the maximum possible score was 1.00. The mixture of the two colors was ~25% for score 0.25, ~50% for score 0.50, and ~75% for score 0.75.

**Table 1:** Algorithm of the chewing efficiency analysis.

(1) Divide the photo into squares of 20 × 20 pixels each.
(2) For each square:
(a) For each pixel in the square:
(i) Get the R(ed), G(reen), B(lue) values of the color of the pixel. (ii) Calculate the 3-dimensional spatial distance of the pixel’s color and the reference colors Blue, Magenta, Gray, and White, by adding up the absolute difference between the R, G, and B values of both colors being compared. (iii) Classify the pixel as Blue, Magenta, Gray, or White based on the shortest spatial distance.
(b) Assign a value to the square, based on the categorized pixels in the square: (i) None—If >50% of pixels is White, the square is not counted (ii) 0.00—If >75% of pixels is Blue (iii) 0.00—If >75% of pixels is Magenta (iv) 0.50—If >25% of pixels is Blue and >25% of pixels is Magenta (v) 0.75—If >25% of pixels is Gray and >25% of pixels is Blue (vi) 0.75—If >25% of pixels is Gray and >25% of pixels is Magenta (vii) 1.00—If >50% of pixels is Gray
(3) Calculate the average of the scored squares as the final Chewing Efficiency Analysis score. The value is between 0.00 and 1.00.

### Dentition

All oral examinations took place by one dentist, experienced in gerodontology, who was blind to the MMSE score at the moment of the examination. The standardized examination took place at the outpatient clinics and nursing homes, with a mouth mirror and head light (Black Diamond, UT, USA). The oral examination included counting of the number of present teeth and the number of occluding pairs (Käyser, 1990). For the number of occluding pairs, a natural premolar contact between the upper and lower jaw counted as one occluding pair and a full natural molar contact as two occluding pairs, resulting in a minimum of 0 occluding pairs and a maximum of 14 occluding pairs. Furthermore, the presence of prosthesis was recorded. At least three occluding pairs or the presence of replacing prosthesis were considered acceptable for chewing ability (Gerritsen et al., 2013).

### Statistical Analysis

The data were analyzed with IBM Statistics SPSS 26 (SPSS Inc., Chicago, IL, USA). The continuous variables were expressed as means and standard deviations (SD) for parametric data and as medians and interquartile ranges (IQR) for nonparametric data. The categorical variables were presented as numbers and percentages. Normality of distribution was assessed with the Shapiro–Wilk test. The means of two continuous dependent variables of two categorical independent variables were compared with the independent *t*-test for parametric data and the Mann–Whitney *U* test for nonparametric data. The means of two categorical variables were compared with the Pearson chi-square test.

Univariate and multivariate linear regression analyses were performed to identify predictors for Chewing Efficiency Analysis score. Variables with *p* < 0.10 in the univariate regression were included in the multivariate backward stepwise analysis to identify the predictors. *p* < 0.05 was considered statistically significant. Multicollinearity was identified with the correlation matrix (*r* < 0.90), variance inflation factor (>10), and tolerance statistics (<0.10).

## Results

### Participants

**Figure 1:**
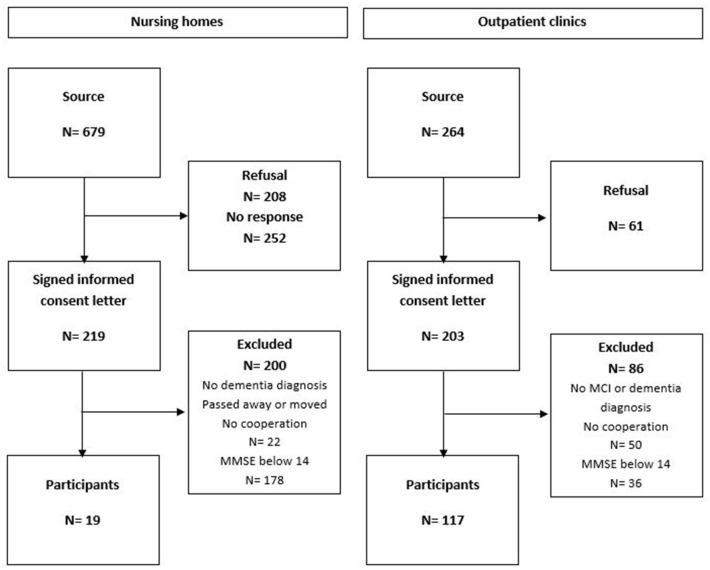
Flow chart of participant inclusion.

Of the individuals visiting the hospitals, 264 were approached for participation, 203 signed the informed consent letter, and 117 were included in this study (Figure 1). The main reason for refusal was the expected burden for the participants, and the main reasons for exclusion were the absence of an MCI or dementia diagnosis or an MMSE score lower than 14. For 679 nursing home residents, an informed consent letter was sent to the legal representatives. Following this, 252 did not respond and 208 refused participation. The main reason for refusal was the expected burden for the participants, and the main reason for exclusion was an MMSE score lower than 14. Nineteen nursing home residents remained. In total, 136 persons were included in this study.

### Descriptive Data

Table 2 shows the descriptive data of the 136 participants. The mean age of the participants was 82.1 (SD 5.8) years, and 74 (=54.4%) were female. The median number of educational years was 11.0 (IQR 9.0–12.0). The majority of the participants were home-dwelling: 117 (86.0%).

**Table 2:** Descriptive data.

Variable	All (*n* = 135)	Full prosthesis upper (*n* = 64)	No full prosthesis (e.g., dentate) upper (*n* = 72)
Age, mean (SD) years	82.1 (5.8)	84.1 (5.7)***	80.4 (5.5)***
Gender, female (%)	74 (54.4%)	39 (60.9%)	35 (48.6%)
Education, median (IQR) years	11.0 (9.0–12.0)	9.5 (8.0–11.8)***	12.0 (10.0–14.0)***
Living environment, number home dwelling (%)	117 (86.0%)	49 (76.6%)**	68 (94.4%)**
Cognition, median (IQR) MMSE score	23.0 (18.0–26.0)	22.0 (18.0–26.0)	23.0 (19.0–27.0)
Present teeth, median (IQR)	13.0 (0.0–23.0)	0.0 (0.0–6.0)***	22.5 (18.3–26.0)***
Occluding pairs, median (IQR)	0.0 (0.0–7.0)	n/a	6.0 (2.0–9.0)
Oral function
0–2 OP and no prosthesis, *N* (%)	7 (5.1%)	n/a	7 (9.7%)
0–2 OP and prosthesis, *N* (%)	71 (52.2%)	58 (90.6%)	13 (18.1%)
3–5 OP, *N* (%)	13 (9.6%)	n/a	13 (18.1%)
≥6 OP, *N* (%)	38 (27.9%)	n/a	38 (52.8%)
Chewing efficiency, median (IQR) score	0.46 (0.14–0.59)	0.15 (0.09–0.38)***	0.57 (0.46–0.68)***

IQR, interquartile range; MMSE, Mini Mental State Examination; N, number; **p < 0.01, ***p < 0.001, OP, occluding pairs; SD, standard deviation.

The participants had a median MMSE score of 23.0 (IQR 18.0–26.0) and a median Chewing Efficiency Analysis score of 0.46 (IQR 0.14–0.59). The median number of teeth was 13.0 (IQR 0.0–23.0), and the median number of occluding pairs was 0.0 (IQR 0.0–7.0). Sixty-four (47.4%) of the participants wore full prosthesis in the upper jaw. Seventy-one (52.2%) of the participants had 0–2 occluding pairs and wore full or partial prostheses. Thirty-eight (27.9%) participants had six or more occluding pairs.

On average, the group with full prosthesis in the upper jaw (FPU) was significantly older (mean 84.1, SD 5.6) than the group without FPU, or a dentate upper jaw (mean 80.4, SD 5.5), *T*-test (df 134) = −3.93, *p* = 0.000. The groups were not significantly different concerning gender, $\chi_{\left(1\right)}^{2}$ = 2.08, *p* = 0.150. In addition, the group with FPU included significantly more nursing-home residents than the group without FPU did, $\chi_{\left(1\right)}^{2}$ = 9.0, *p* = 0.003.

The group with FPU had no occluding pairs and less present teeth than the group without FPU. The MMSE score was not significantly different for the group with FPU (median 22.0, IQR 18.0–26.0) and the group without FPU (median 23.0, IQR 19.0–27.0). The group with FPU had a significantly lower chewing analysis score (median 0.15, IQR 0.09–0.38) than the group without FPU (median 0.57, IQR 0.46–0.68), *U* = 532.0, *p* = 0.000.

### Main Results

Table 3 shows the predictors of Chewing Efficiency Analysis score in univariate linear regression analysis. The variables age, gender, cognition, full prosthesis upper jaw, number of present teeth, and number of occluding pairs were eligible for the multivariate regression model (*p* < 0.10). The covariate education was excluded (*p* = 0.134).

**Table 3:** Predictors of the Chewing Efficiency Analysis score in univariate linear regression analysis.

Variable	*R*	*R*^2^	B	*t*	*p*	95% CI Lower	95% CI Upper
Age	0.35	0.12	-0.02	-4.30	0.000	-0.02	-0.01
Gender	0.16	0.03	0.08	1.89	0.061	-0.00	0.17
Education	0.15	0.02	0.01	1.51	0.134	-0.00	0.03
Cognition	0.18	0.03	0.01	2.10	0.038	0.00	0.02
Full prosthesis upper	0.67	0.44	-0.33	-10.35	0.000	-0.40	-0.27
Present teeth	0.66	0.44	0.02	10.21	0.000	0.01	0.02
Occluding pairs	0.62	0.39	0.04	9.18	0.000	0.03	0.05

*Note: *N* = 135, *B*, unstandardized B; *CI*, confidence interval; *p*, level of significance; *R*, variance; *t*, test value*.

The correlation matrix (Table 4) indicates a very strong, negative correlation between the number of present teeth and the presence of full prosthesis in the upper jaw (*r* = −0.89) and a very strong, positive correlation between the number of present teeth and the number of occluding pairs (*r* = 0.83). To avoid multicollinearity, the number of present teeth was excluded from the multivariate regression model.

**Table 4:** Correlation matrix of the Chewing Efficiency Analysis (CEA) score and the predictors.

Correlations	CEA score	Age	Gender	MMSE	FPU	PT	OP
CEA score	1.00	-0.35	0.15	0.16	-0.68	0.69	0.62
Age	-0.35	1.00	-0.26	-0.35	0.33	-0.32	-0.30
Gender	0.15	-0.26	1.00	0.19	-0.13	0.17	0.12
Cognition (MMSE)	0.16	-0.35	0.19	1.00	-0.08	0.10	0.05
Full prosthesis upper (FPU)	-0.68	0.33	-0.13	-0.08	1.00	-0.89	-0.71
Present teeth (PT)	0.69	-0.32	0.17	0.10	-0.89	1.00	0.83
Occluding pairs (OP)	0.62	-0.30	0.12	0.05	-0.71	0.83	1.00

*Note: *N* = 135*.

In multivariate linear backward regression analysis (Table 5), significant predictors for Chewing Efficiency Analysis score were full prosthesis in the upper jaw (*p* < 0.000) and the number of occluding pairs (*p* < 0.003). The final model explained 52% (*R*^2^ = 0.52) of the variance in Chewing Efficiency Analysis score, *F*_(3,131)_ = 47.23, *p* < 0.000.

**Table 5:** Predictors of the Chewing Efficiency Analysis score in multivariate linear backward regression analysis.

Variable	B	*t*	*p*	95% CI Lower	95% CI Upper
Step 1
(Constant)	0.66			
Age	0.00	-1.21	0.227	-0.01	0.00
Gender	0.01	0.39	0.695	-0.05	0.07
Cognition	0.00	1.21	0.228	0.00	0.01
Full prosthesis upper	-0.23	-5.26	0.000	-0.31	-0.14
Occluding pairs	0.02	3.06	0.003	0.01	0.03
Step 2
(Constant)	0.68			
Age	0.00	-1.31	0.193	-0.01	0.00
Cognition	0.00	1.27	0.207	0.00	0.01
Full prosthesis upper	-0.23	-5.29	0.000	-0.31	-0.14
Occluding pairs	0.02	3.08	0.003	0.01	0.03
Step 3
(Constant)	0.89			
Age	0.00	-1.87	0.064	-0.01	0.00
Full prosthesis upper	-0.23	-5.30	0.000	-0.31	-0.14
Occluding pairs	0.02	3.01	0.003	0.01	0.03

*B, unstandardized B; *CI*, confidence interval; *p*, level of significance; *R*, variance; *t*, test value. R^2^ = 0.53 and *F* = 28.64 (*p* < 0.000) for Step 1. ΔR^2^ = 0.00 and *F* = 35.99 (*p* < 0.000) for Step 2. ΔR^2^ = 0.01 and *F* = 47.23 (*p* < 0.000) for Step 3. *N* = 135*.

## Discussion

The aim of this cross-sectional observational study was to examine global cognitive functioning and dentition as predictors for chewing efficiency in older people with MCI or dementia. One of the main findings was that full prosthesis in the upper jaw and number of occluding pairs were significant predictors for Chewing Efficiency Analysis score. After adjusting for these two predictors and age, the MMSE score was not a significant predictor.

In the current study, a strong, positive correlation was found between the chewing efficiency and the number of teeth present. In a study of Ikebe et al. (2011), a comparable correlation (*r* = 0.57) was found between masticatory performance and the number of residual teeth in a sample of 1,288 community-dwelling, independently living people over the age of 60 years. Furthermore, there was a moderate, negative correlation between age and chewing efficiency in the current study, while there was a weak, negative correlation between age and masticatory performance in the study by Ikebe et al. (2011). In the latter study, gummy jellies were used to measure masticatory performance. Previous research showed a decline in masticatory performance with age, although this decline was not gradual, but stronger in older age (Lin et al., 2017). With aging, the integrity of the white matter of the brain might show a decline, resulting in a lower signal transmission between brain regions (Malykhin et al., 2011). Consequently, neural systems, including the frontal lobe, the striatum, and the cerebellum, involved in mastication among others, may show an age effect (Sesay et al., 2000). Therefore, a reduced masticatory function as part of general aging effect can be expected (Taubert et al., 2020). Additionally, the study by Ikebe et al. (2011) showed that masticatory performance was significantly associated with occlusal bite force and stimulated salivary flow rate. However, the level of global cognitive functioning was not reported.

A study by Kim et al. (2017) involved the level of cognitive impairment, measured with the MMSE of Dementia Screening (MMSE-DS), and found a significant association with chewing efficiency, measured with a color-changing gum. Furthermore, the study by Kim et al. (2017) reported that participants with a lower chewing efficiency had poorer nutritional status. At the same time, no oral health examination could be done and no correlation could be determined with dentition.

Lexomboon et al. (2012) examined chewing efficiency and tooth loss and the association with cognitive impairment in older people. The study found a significant relation between self-reported chewing efficiency and cognitive function, after adjusting for age, gender, and level of education. The association between tooth loss and cognition was mainly explained by age and education. Both the study by Lexomboon et al. (2012) and the current study found that global cognition had a stronger correlation with age than with the number of teeth present. The difference in chewing efficiency could be explained by the measuring methods of both studies: the study by Lexomboon et al. (2012) used self-reported dental status and chewing difficulty, while the current study included a dental examination by a dentist and a standardized chewing efficiency test. Another explanation might be that people with mild to moderate cognitive impairment still have sufficient capacity for compensation of impairments, such as a reduction in mastication, while this might not be the case in people with severe dementia (Henskens et al., 2018).

People with FPU had a lower chewing efficiency score than people without FPU in the present study, which might be explained by an actual lower chewing efficiency or, alternatively, by the sticking of the chewing gum to the prosthesis. Furthermore, people with prosthesis might also take more time and chewing cycles to achieve the same chewing result (Gonçalves et al., 2014). In addition, people with full prosthesis make smaller vertical and lateral chewing movements than people with natural dentition do (Gonçalves et al., 2014).

Although it is known that age is a risk factor for cognitive decline and tooth loss (Beydoun et al., 2014; Kossioni et al., 2018; Müller et al., 2017), it could also be a risk factor for deterioration in chewing efficiency (Lin et al., 2017; Avivi-Arber and Sessle, 2018).

### Strengths and Limitations

One of the major limitations of this study was that the chewing gum stuck to the prosthesis. Therefore, it was decided to split the data into a group with full prosthesis in the upper jaw and a group without full prosthesis in the upper jaw, or a dentate upper jaw.

Furthermore, the MMSE does not specifically test loss of spatial memory and learning capacity, although these cognitive functions might be reduced in relation to reduced masticatory activity (Weijenberg et al., 2011). However, the MMSE tests memory (among other cognitive functions), which is related to the hippocampus as part of a complex memory network. The hippocampus showed degeneration in animal studies after reduced masticatory activity (Yamamoto and Hirayama, 2001; Watanabe et al., 2002; Tsutsui et al., 2007). Another limitation of this study was the MMSE cutoff score of 14. Consequently, people with severe cognitive impairment were excluded, while it could be hypothesized that the chewing efficiency in this group could be affected the most.

At the same time, the MMSE cutoff score of 14 can be seen as a strength, because the participants could be expected to understand the instructions (Hadjistavropoulos et al., 2010). The MMSE is a widely used instrument to screen global cognitive functioning, which makes comparison with other studies and interpretation easier (Folstein et al., 1975). In addition, the non-response rate of the current study was clearly described.

Another strength was that all participants were examined by the same dentist, who had experience in geriatric dentistry and was not informed about the MMSE score of the participants during the dental examination. A structured dental examination took place, instead of using questionnaires to assess the number of teeth present, the number of occluding pairs, and the presence of prostheses. Moreover, the chewing efficiency was measured objectively with a standardized two-colored gum and analyzed with an algorithm programmed specifically for this purpose.

### Generalizability

The current study included a sample of participants with mild to moderate cognitive impairment or mild to moderate dementia, living in the community (86%) or nursing homes (14%) in the Netherlands. Following the inclusion criteria of MCI or dementia and the MMSE cutoff score of 14 or higher, it did not include people without cognitive impairment or people with severe cognitive impairment.

### Clinical Relevance

This study showed a strong, positive correlation between the chewing efficiency and the number of teeth present and the number of occluding pairs, indicating that their maintenance is important to maintaining a good chewing efficiency. Tooth loss can lead to chewing difficulties and food deficiencies, affecting general health (Kossioni et al., 2018). The oral health-related quality of life (OHRQoL) and chewing efficiency might be restored partially with removable prostheses in people with cognitive impairment or dementia (Campos et al., 2018).

Oral health is important in maintaining food intake, general health, and quality of life (Niesten et al., 2012; Furuta et al., 2013; Peres et al., 2019; van de Rijt et al., 2019). The maintenance of oral health in older people with cognitive impairment requires specialized care (Janssens et al., 2018b; Marchini et al., 2019), including oral health-care assistance by formal and informal caregivers, knowledge about oral health, general health, and cognitive impairment, as well as a multidisciplinary collaboration (Delwel et al., 2018; Jablonski et al., 2018; Janssens et al., 2018a).

### Future Research

For future studies, it is recommended to use an objective measurement for the assessment of chewing efficiency (Weijenberg et al., 2011). For this purpose, a two-colored chewing gum could be used, which is a validated method to evaluate chewing efficiency (Weijenberg et al., 2013). An advantage of the chewing gum is the sweet taste, which could encourage cooperation of study participants. Our clinical observation was that the chewing gum could stick to full prosthesis in the upper jaw. At the same time, Silva et al. specifically studied the reliability of chewing gum in participants with full prosthesis in the upper and lower jaw and concluded that it can be used reliably to assess mastication in this group (Silva et al., 2018). An alternative method to evaluate chewing efficiency in participants with prosthesis is the use of red and blue wax (Speksnijder et al., 2009) or gummy jellies (Ikebe et al., 2011). The Chewing Efficiency Analysis software that was used in the current study is also suitable for the analysis of other materials with contrasting colors (see algorithm in Table 1). In addition to a mixing ability test, the evaluation of maximum voluntary bite force is important to assess the level of functioning of the masticatory system (Speksnijder et al., 2009; Ikebe et al., 2011; Weijenberg et al., 2011).

For the screening of global cognitive functioning, the MMSE is suitable and widely used (Nilsson et al., 2014). In order to study the relation between chewing and cognitive functioning, more specific cognitive tests seem interesting, such as tests for spatial memory and learning capacity (Teixeira et al., 2014; Weijenberg et al., 2018). Moreover, a multifactorial approach is needed, including age, socioeconomic status, cognition, dentition, general health, nutrition, chewing efficiency, inflammatory factors, and stress levels (Azuma et al., 2017; Weijenberg et al., 2018).

## Conclusion

Better mastication is associated with a higher number of occluding pairs. Moreover, full prosthesis in the upper jaw is related to a lower chewing efficiency, compared to natural dentition in the upper jaw. Furthermore, global cognitive functioning is not associated with mastication in older people with MCI or mild to moderate dementia, after adjusting for age and dentition. This might be explained by sufficient capacity for compensation of reduced mastication in this group.

## Data Availability Statement

The datasets generated for this study are available on request to the corresponding author.

## Ethics Statement

The study protocol was reviewed and approved by the Medical Ethics Review Committee of the Amsterdam UMC (approval number NL 43861.029.13). The participants or their legal representatives provided written informed consent for participation in this study.

## Author Contributions

ES, FL, and SD designed the study. JM developed the Chewing Efficiency Analysis software. SD collected and analyzed the data. SD and DP drafted the manuscript. All authors contributed to the article and approved the submitted version.

## Conflict of Interest

The authors declare that the research was conducted in the absence of any commercial or financial relationships that could be construed as a potential conflict of interest.
